# Metabolism of the EGFR tyrosin kinase inhibitor gefitinib by cytochrome P450 1A1 enzyme in EGFR-wild type non small cell lung cancer cell lines

**DOI:** 10.1186/1476-4598-10-143

**Published:** 2011-11-23

**Authors:** Roberta R Alfieri, Maricla Galetti, Stefano Tramonti, Roberta Andreoli, Paola Mozzoni, Andrea Cavazzoni, Mara Bonelli, Claudia Fumarola, Silvia La Monica, Elena Galvani, Giuseppe De Palma, Antonio Mutti, Marco Mor, Marcello Tiseo, Ettore Mari, Andrea Ardizzoni, Pier Giorgio Petronini

**Affiliations:** 1 Department of Experimental Medicine, Unit of Experimental Oncology, University of Parma, Parma, Italy; 2 Italian Workers' Compensation Authority (INAIL) Research Center at the University of Parma, Italy; 3 Department of Clinical Medicine, Nephrology and Health Science, Laboratory of Industrial Toxicology, University of Parma, Italy; 4 Department of Experimental and Applied Medicine, Section of Occupational Medicine and Industrial Hygiene, University of Brescia, Italy; 5 Pharmaceutical Department, University of Parma, Parma, Italy; 6 Division of Medical Oncology, University Hospital of Parma, Italy; 7 AstraZeneca Medical Department, Basiglio, Milan, Italy

**Keywords:** Lung cancer, EGFR, gefitinib, metabolism, CYP1A1

## Abstract

**Background:**

Gefitinib is a tyrosine kinase inhibitor (TKI) of the epidermal growth factor receptor (EGFR) especially effective in tumors with activating EGFR gene mutations while EGFR wild-type non small cell lung cancer (NSCLC) patients at present do not benefit from this treatment.

The primary site of gefitinib metabolism is the liver, nevertheless tumor cell metabolism can significantly affect treatment effectiveness.

**Results:**

In this study, we investigated the intracellular metabolism of gefitinib in a panel of EGFR wild-type gefitinib-sensitive and -resistant NSCLC cell lines, assessing the role of cytochrome P450 1A1 (CYP1A1) inhibition on gefitinib efficacy. Our results indicate that there is a significant difference in drug metabolism between gefitinib-sensitive and -resistant cell lines. Unexpectedly, only sensitive cells metabolized gefitinib, producing metabolites which were detected both inside and outside the cells. As a consequence of gefitinib metabolism, the intracellular level of gefitinib was markedly reduced after 12-24 h of treatment. Consistent with this observation, RT-PCR analysis and EROD assay showed that mRNA and activity of CYP1A1 were present at significant levels and were induced by gefitinib only in sensitive cells. Gefitinib metabolism was elevated in crowded cells, stimulated by exposure to cigarette smoke extract and prevented by hypoxic condition. It is worth noting that the metabolism of gefitinib in the sensitive cells is a consequence and not the cause of drug responsiveness, indeed treatment with a CYP1A1 inhibitor increased the efficacy of the drug because it prevented the fall in intracellular gefitinib level and significantly enhanced the inhibition of EGFR autophosphorylation, MAPK and PI3K/AKT/mTOR signalling pathways and cell proliferation.

**Conclusion:**

Our findings suggest that gefitinib metabolism in lung cancer cells, elicited by CYP1A1 activity, might represent an early assessment of gefitinib responsiveness in NSCLC cells lacking activating mutations. On the other hand, in metabolizing cells, the inhibition of CYP1A1 might lead to increased local exposure to the active drug and thus increase gefitinib potency.

## Background

Gefitinib is an orally active, selective EGFR TKI used in the treatment of patients with advanced-NSCLC carrying activating EGFR mutations [1]. In fact, it is well established that gefitinib is more active in some patient subgroups, such as Asians, females, never smokers and adenocarcinoma histotypes which have a higher probability of harbouring activating mutations in the tyrosine kinase domain, the most frequent being L858R in exon 21 and Del (746-750) in exon 19 [1]. As a consequence most of the NSCLCs containing wild-type EGFR receptor are excluded and hence the role of gefitinib for the treatment of NSCLC is limited. However, some studies have shown that patients without mutations responded to gefitinib with response rates reaching 6.6% [2,3]. In addition to cancer cell genomic determinants of sensitivity, some pharmacokinetic parameters may also play a role in the variable response to gefitinib and other TKIs [4].

When administered at 250 mg/day, gefitinib is 60% orally absorbed and 90% plasma protein-bound [5,6]. The very high distribution volume of gefitinib (1400 litres) clearly indicates that the drug is extensively distributed in tissues such as liver, kidney, gastrointestinal tract, lung and in tumors [7]. A tendency to accumulate in the lung was observed with concentrations 10 times higher than in plasma [8]. We have recently demonstrated in NSCLC cell lines that the uptake of gefitinib is an essentially active process leading to intracellular gefitinib concentrations more than two hundred times higher than outside the cells [9].

There are few data on gefitinib intracellular metabolism in tumors, the majority of the available data concerns liver metabolism. *In vitro *and *in vivo *studies indicate that in the liver gefitinib is mainly metabolized by cytochrome P450-dependent (CYP) activities, including CYP3A4, CYP3A5 and CYP2D6 [10-12]. The main metabolic pathway characterized by using human liver microsomes include morpholine ring opening, O-demethylation of the methoxy-substituent on the quinazoline ring structure and oxidative defluorination of the halogenated phenyl group [13,14].

A study investigating the contribution of individual CYPs to gefitinib metabolism demonstrated that gefitinib disappeared with similar clearance when incubated with CYP3A4 or CYP2D6 enzymes, less efficiently with CYP3A5 or CYP1A1, whereas CYP1A2 and CYP1B1 were not involved in the metabolism of the drug [12]. Incubation with CYP3A4 and to a lesser extent CYP3A5, produced a similar range of metabolites as that produced by liver microsomes [11], but the main plasma metabolite, the O-desmethyl derivative present at plasma concentrations similar to gefitinib [10], was formed predominantly through the CYP2D6 enzyme.

CYP1A1 is one of the three members of the CYP1 family mainly expressed in extra-hepatic tissue, involved in the metabolism of a large number of xenobiotics as well as a small number of endogenous substrates [15]. Being expressed at a significant level in human lung [16], it might play a role in the metabolism of gefitinib by lung tumor cells and its activity might be involved in the variability of the drug response.

In experiments using lung microsomes, CYP1A1 was shown to produce significant amounts of the *para*-hydroxyaniline metabolite derived from oxidative defluorination of gefitinib. Hydroxyaniline metabolites produced by CYP1A1 can be oxidized to reactive quinone-imine derivatives that form adducts with nucleophilic groups of macromolecules or GSH and may be related to clinically relevant hepatotoxicity or interstitial lung disease [8].

Both mRNA and protein CYP1A1 levels in human lung are greatly induced by tobacco smoke [17] and it has been reported that lung microsomes from smokers may generate 12 times more gefitinib-derived reactive metabolites as compared to non-smokers [8].

The present study was designed to investigate gefitinib metabolism in a panel of EGFR wild-type NSCLC cell lines either sensitive or resistant to gefitinib. Our objective was to define a possible potential role of gefitinib metabolism in early evaluation of tumor response to gefitinib, to analyze conditions or factors that can alter tumor gefitinib metabolism and to test the effect of CYP1A1 inhibition on gefitinib efficacy.

## Methods

### Cell culture

The human NSCLC cell lines H322, Calu-3, H292, H460, H1299, A549, Calu-1 and SKLU-1 were cultured as recommended. Cell lines obtained from American Type Culture Collection (Manassas, VA, USA) were immediately expanded and frozen. Every four months all the cell lines were restarted from a frozen vial of the same batch of cells and no additional authentication was done in our laboratory. All cells were maintained under standard cell culture conditions at 37°C in a water-saturated atmosphere of 5% CO_2 _in air. As previously reported [18] cells showing in proliferation assays IC_50 _for gefitinib < 1 μM were considered sensitive (H322, H292, Calu-3) and cell lines with IC_50 _> 8 μM (H460, H1299, A549, Calu-1 and SKLU-1) were considered resistant.

### Hypoxia

Hypoxic conditions (0.5%O_2_) were established by placing the cells in a tissue culture incubator (Binder GmbH, Tuttlingen, Germany) with controlled O_2 _levels.

### Preparation of cigarette smoke extract (CSE)

CSE preparation was made according to Carp and Janoff [19], with slight modifications. Briefly, one cigarette without filter was combusted using a modified syringe-driven apparatus and the smoke was bubbled through 50 ml of serum-free cell culture medium. This solution, considered to be 100% CSE, was filtered diluted with medium and applied to cell cultures within 30 min of preparation.

### CYP1A1 genotyping

Genomic DNA was isolated using a PureGene DNA purification system (GENTRA SYSTEMS, Minneapolis, MN) and both the *rs 4646903 *(3798 T > C, variant allele CYP1A1*2A) and the *rs 1048943 *(2454 A > G, variant allele CYP1A1*2C) polymorphisms of the *CYP1A1 *gene that were characterized according to previously published methods, with minimal changes [20,21]. All the tested cell lines carried a *wild type *homozygous genotype for both the polymorphisms.

### Drug treatment

Gefitinib (ZD1839/Iressa) and metabolites [morpholine ring-opening (M537194, M1), oxidative defluorinated (M387783, M2) and O-desmethyl (M523595, M3) derivatives] were kindly provided by AstraZeneca. α-naphthoflavone (α-NAP) was from Sigma Aldrich (St. Louis, MO, USA).

Cetuximab, erlotinib and lapatinib were from inpatient pharmacy. RAD001 and NVP-BEZ235 were provided by Novartis Institutes for BioMedical Research (Basel, Switzerland). Wortmannin, PD98059 and U0126 were from Sigma-Aldrich (St. Louis, MO).

### Uptake measurements

[^3^H]gefitinib uptake by cells was determined as described recently [9].

### Liquid chromatography tandem mass spectrometry (LC-MS/MS)

For LC-MS/MS analysis, the medium samples were treated with ethyl acetate, dried under nitrogen and refilled with methanol and aqueous formic acid (0.1 M), while the ethanolic extracts were diluted with aqueous formic acid (0.1 M).

LC analyses were carried out with an Agilent HP 1100 pump coupled with a API4000 triple-quadrupole mass spectrometer (Sciex, Concord, Canada) equipped with a TurboIonSprayTM interface and configured in Selected Reaction Monitoring (SRM) mode. Chromatography was performed on a Synergi Hydro-RP column (5 × 2.0 mm i.d., 2 μm; Phenomenex) using variable proportions of 10 mM aqueous formic acid and methanol/acetonitrile (95/5, v/v) mixture as the mobile phase.

The analytes were ionized in positive ion mode and the following SRM transitions were monitored: m/z 447 ([M+H]+) → 128 for Gefitinib; m/z 421 ([M+H]+) → 320 for Metabolite 1; m/z 445 ([M+H]+) → 128 for Metabolite 2; m/z 433 ([M+H]+) → 128 for Metabolite 3 and m/z 394 ([M+H]+) → 336 for Internal Standard. Erlotinib was used as Internal Standard.

### Determination of cell growth

Cell number and viability were evaluated by cell counting, crystal violet staining and MTT colorimetric assay as previously described [18].

### Western blot analysis

Procedures for protein extraction, solubilization, and protein analysis by 1-D PAGE are described elsewhere [22]. Anti-EGFR, anti-phospho-EGFR (tyr1068), anti-phospho-p44/42 MAPK, anti-p44/42 MAPK, anti-phospho-AKT (Ser473), anti-AKT and anti-actin were from Cell Signaling Technology (Beverly, MA, USA).

### Real-Time RT-PCR

Total RNA was isolated by the TRIzol^® ^reagent (Invitrogen, Carlsbad, CA, USA) and reverse-transcribed as previously described [23]. The transcript levels of *CYP1A1, CYP1A2, CYP2D6, CYP3A4 *and *CYP3A5 *genes were assessed by Real-Time qRT-PCR on an iCycler iQ Multicolor RealTime PCR Detection System (Bio-Rad, Hercules, CA, USA). Primers and probes included: CYP1A1-F (5'-TCCAAGAGTCCACCCTTCC-3'), CYP1A1-R (5'-AAGCATGATCAGTGTAGGGATCT-3'), CYP1A1-probe (5'-FAM CAGCCACC 3'DQ); CYP1A2-F (5'-GGCTTCTACATCCCCAAGAA-3'), CYP1A2-R (5'-CAGCTCTGGGTCATGGTTG-3'), CYP1A2-probe (5'-FAM CAGTGGCA 3'DQ); CYP2D6-F (5'-CTTCCAAAAGGCTTTCCTGA-3'), CYP2D6-R (5'-CAGGTCATCCTGTGCTCAGTT-3'), CYP2D6-probe (5'-FAM GCTGGATG 3'DQ); CYP3A4-F (5'-GATGGCTCTCATCCCAGACTT-3'), CYP3A4-R (5'AGTCCATGTGAATGGGTTCC-3'), CYP3A4-probe (5'-FAM TCCTCCTG 3'DQ); CYP3A5-F (5'-TGCCCAGTATGGAGATGTATTG-3'), CYP3A5-R (5'-GCTGTAGGCCCCAAAGATG-3'), CYP3A5-probe (5'-FAM GGAAGCAG 3'DQ); PGK1-F (5'-GGAGAACCTCCGCTTTCAT-3'), PGK1-R (5'-CTGGCTCGGCTTTAACCTT-3'), PGK1-probe (5'-FAM GGAGGAAG 3'DQ); RPL13-F (5'-ACAGCTGCTCAGCTTCACCT-3'), RPL13-R (5'-TGGCAGCATGCCATAAATAG-3'), RPL13-probe (5'-FAMCAGTGGCA3'DQ); HPRT-F (5'-TGACCTTGATTTATTTTGCATACC-3'), HPRT-R (5'CGAGCAAGACGTTCAGTCCT-3'), HPRT-probe (5'-FAM GCTGAGGA 3'DQ).

The amplification protocol consisted of 15 min at 95°C followed by 40 cycles at 94°C for 20 s and at 60°C for 1 min.

The relative transcript quantification was calculated using the *geNorm *algorithm for Microsoft Excel™ after normalization by expression of the control genes [*phosphoglycerate kinase1 *(*PGK1*)*, ribosomal protein L13 *(*RPL13*) and *hypoxanthine-guanine*-*phosphoribosyltransferase *(*HPRT*)] and expressed in arbitrary units (a.u.).

### EROD assay

The CYP1A1 ethoxyresorufin-O-deethylase activity (EROD) was determined in intact cells as described by Kennedy and Jones [24] with 5 μM ethoxyresorufin in growth medium as substrate in the presence of 1.5 mM salicylamide, to inhibit conjugating enzymes. The assay was performed at 37°C. The fluorescence of resorufin generated by the conversion of ethoxyresorufin by CYP1A1 was measured first, immediately after addition of reagents and then every 10 min for 60 min at 37°C in a Tecan infinite 200 fluorescence plate reader with excitation of 530 nm and emission at 595 nm. A standard curve was constructed using resorufin.

### RNA interference assay

Cells were transfected with Invitrogen Stealth™ siRNA against CYP1A1 (1:1:1 mixture of ^#^102535, ^#^102537 and ^#^175787) or scramble negative control (1:1:1 mixture of ^#^12935-200, ^#^12935-300 and ^#^12935-400) with a final concentration of 30 nM. The transfection was carried out according to the Invitrogen forward transfection protocol for Lipofectamine™ RNAiMAX transfection reagent. After 48 h of transfection, the transfection medium was aspirated and replaced with exposure medium.

### Statistical analysis

The statistical analyses were carried out using GraphPad Prism version 5.00 software (GraphPad Software Inc., San Diego, CA). Results are expressed as mean values ± standard deviations (SD) for the indicated number of independent measurements. Differences between the mean values recorded for different experimental conditions were evaluated by Student's t-test, and P values are indicated where appropriate in the figures and in their legends. A P value < 0.05 was considered as significant.

## Results

### Intracellular and extracellular levels of gefitinib in sensitive and resistant NSCLC cell lines

**Figure 1:**
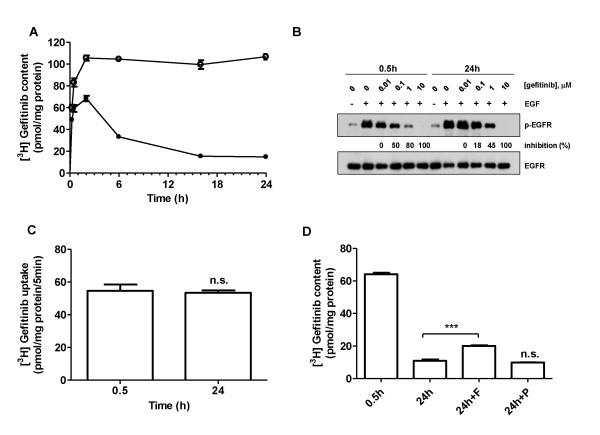
**Intracellular content of gefitinib in NSCLC cell lines and its effect on EGFR autophosphorylation**. A) Time course of 0.1 μM [^3^H]gefitinib accumulation in H322 (●) and H1299 (○) cell lines. Each point represents the mean ± SD of four independent determinations. B) H322 cells were incubated for 0.5 h and 24 h with the indicated extracellular concentrations of gefitinib before stimulation with 0.1 μg/ml EGF for 5 min. Western blot analysis was performed by using monoclonal antibodies directed to p-EGFR(p-Tyr1068) and to EGFR. The immunoreactive spots were quantified by densitometric analysis, ratios of phosphotyrosine/total EGFR were calculated and values are expressed as percentage of inhibition versus control. The experiment, repeated three times, yielded similar results. C) H322 cells were incubated with 0.1 μM gefitinib for 0.5 h or 24 h and then the initial rate (5 min) of 0.1 μM [^3^H]gefitinib uptake was measured. Each bar represents the mean ± SD of four independent determinations. D) H322 cells were exposed to 0.1 μM [^3^H]gefitinib for 0.5 h and 24 h, in the absence or in the presence of 10 μM Fumetrimorgin C (F) or PSC833 (P) and then intracellular gefitinib content was determined. Values given are the means (± SD) of three independent determinations (***P < 0.001).

In the first part of the study we evaluated the accumulation kinetics of 0.1 μM radiolabeled gefitinib in H322-sensitive and H1299-resistant cell lines during 24 h of treatment. Figure 1A shows a progressive decrease of the level of intracellular radiolabeled gefitinib only in the sensitive cell line. The decrease was detectable starting from 6 h of treatment, reaching a minimum level after 16 h. Similar results were obtained with a higher (1 μM) gefitinib concentration (not shown).

We then analyzed the effect of the intracellular gefitinib level on EGFR autophosphorylation in H322 cells. As reported in Figure 1B after 0.5 h, gefitinib inhibited EGFR autophosphorylation by around 50% and 80% at doses of 0.1 μM and 1 μM respectively; after 24 h these inhibitions were significantly reduced indicating a correlation between the intracellular gefitinib level and the inhibition of EGFR phosphorylation, confirming our previous results [9].

In an attempt to investigate whether the fall in intracellular gefitinib could be related to a lower influx, an enhanced efflux or metabolism of the drug, we firstly measured 5 min of [^3^H]gefitinib uptake in H322 cells treated with gefitinib for 0.5 h and 24 h (Figure 1C) and the level of intracellular gefitinib in the presence of inhibitors of specific efflux transporters (Figure 1D). As shown in Figure 1C, the initial rate of [^3^H]gefitinib uptake at 0.5 h and at 24 h was similar, suggesting that in the presence of an extracellular fixed concentration of drug, its influx is constant over time. Since it has been reported that gefitinib interacts with ABCG2 and to a lesser extent with ABCB1 [25], the intracellular levels of the radiolabeled drug were determined after dosing cells with the respective inhibitors Fumitremorgin C (Sigma Aldrich) and PSC833 (kindly provided by Novartis). We demonstrated only a slight increase in gefitinib content at 24 h in the presence of Fumitremorgin C (inhibition of ABCG2), whereas the inhibition of ABCB1 pump was ineffective (Figure 1D).

**Figure 2:**
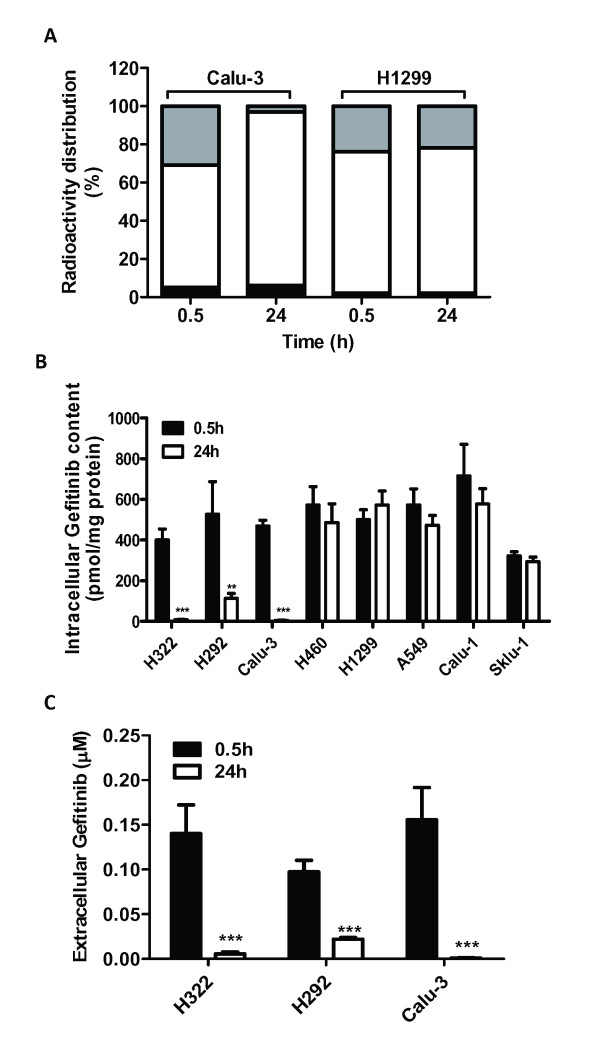
**Intracellular and extracellular level of gefitinib in NSCLC cell lines**. A) Radioactivity distribution in Calu-3 and H1299 cells incubated with 0.1 μM [^3^H]gefitinib for 0.5 h or 24 h. The amount of radioactivity was determined in intracellular (gray), extracellular (white) and intracellular macromolecule-linked (black) compartments. Values are given as percentage versus the total amount of radioactivity. B) Intracellular level and C) extracellular concentration of gefitinib in a panel of NSCLC cell lines evaluated by LC-MS/MS. Gefitinib-sensitive cell lines (H322, H292, Calu-3) and resistant-cell lines (H460, H1299, A549, Calu-1, SKLU-1) were incubated with 0.1 μM gefitinib for 0.5 h and 24 h. Values given are the means (± SD) of four independent determinations (**P < 0.01; ***P < 0.001).

We then analyzed the distribution of radioactivity among intracellular, extracellular and macromolecule-linked compartments in another sensitive, EGFR wild-type cell line (Calu-3) and in resistant H1299 after 0.5 h and 24 h of treatment with radiolabeled gefitinib. As shown in Figure 2A, Calu-3 showed a significant drop in intracellular radioactivity, with a parallel increase in extracellular radioactivity after 24 h of incubation; by contrast, the radioactivity distribution was unchanged between 0.5 h and 24 h in H1299 cells. The amount of radioactivity in the NaOH fraction (macromolecule-linked) was less than 10% in both cell lines.

Since the measured radioactivity may be associated, at least in part, with gefitinib metabolites, the actual amount of gefitinib was monitored intracellularly and in the medium by LC-MS/MS after 0.5 h and 24 h of treatment in a panel of NSCLC cell lines showing either sensitivity (H322, H292, Calu-3) or resistance (H460, H1299, A549, Calu-1, SKLU-1) to the drug.

As shown in Figure 2B, the intracellular level of gefitinib was markedly reduced at 24 h (more than 80%) in all the sensitive cell lines, whereas the resistant ones showed a slight reduction (lower than 20%). Figure 2C shows that in sensitive cell lines, the extracellular level of gefitinib after 24 h of treatment was markedly reduced indicating that the increased radioactivity in the medium at 24 h (Figure 2A) was not due to gefitinib itself but to radiolabeled molecules probably derived from intracellular metabolism of gefitinib and then extruded into the extracellular compartment.

Taken together these results clearly demonstrate that the observed decrease in gefitinib content evident only in sensitive cells was due to a high rates of gefitinib metabolism.

### Production of gefitinib metabolites by NSCLC cell lines and their effect on cell growth and EGFR autophosphorylation

Employing the standards kindly provided by AstraZeneca, we analyzed the appearance of the three main gefitinib metabolites (M1; M2; M3) inside and outside the cells after 0.5, 6 and 24 h of treatment with 0.1 μM gefitinib.

**Figure 3:**
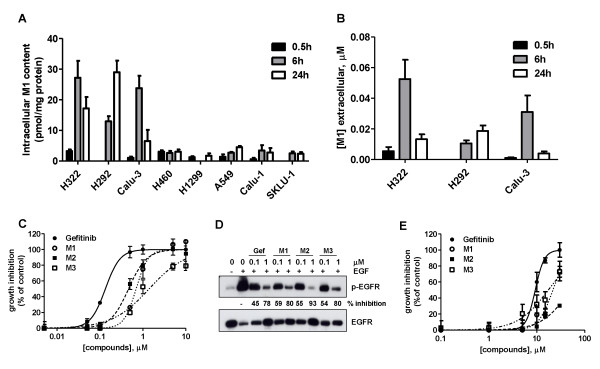
**Production and biological activity of gefitinib metabolites**. Cells were incubated with 0.1 μM gefitinib for 0.5, 6 or 24 h and then the M1 content was determined both in the intracellular (A) and extracellular (B) compartment, through LC-MS/MS analysis. M1 levels calculated for each cell line were expressed as pmol/mg of protein (intracellular) or μM (extracellular). Values given are the means (± SD) of three independent determinations C) H322 cells were exposed for 72 h to different concentrations of gefitinib or its metabolites (ranging from 0.05 to 10 μM) and then cell growth was assessed using crystal violet staining. Data are expressed as percent inhibition of cell proliferation *versus *control cells. The mean values of three independent measurements (± SD) are shown. D) H322 cells were incubated for 0.5 h with the indicated concentrations of gefitinib and metabolites, before stimulation with 0.1 μg/ml EGF for 5 min. Western blot analysis was performed by using monoclonal antibodies directed to p-EGFR(p-Tyr1068) and to EGFR. The immunoreactive spots at each point were quantified by densitometric analysis, ratios of phosphotyrosine/total EGFR were calculated and values expressed as percentage of inhibition versus control. The experiment, repeated three times, yielded similar results. E) H1299 cells were exposed for 72 h to different concentrations of gefitinib or its metabolites (ranging from 1 to 30 μM) and then cell growth was assessed using crystal violet staining. Data are expressed as percent inhibition of cell proliferation *versus *control cells. The mean values of three independent measurements (± SD) are shown.

LC-MS/MS analysis (Figure 3A) showed that the M1 metabolite was present at a very low level in the intracellular compartment, mainly in sensitive cell lines, whereas M2 and M3 were undetectable.

The M1 metabolite was also present in the extracellular compartment (Figure 3B) at concentrations between 0.01 and 0.05 μM only in sensitive cell lines.

We then tested on sensitive and resistant cell lines whether metabolites M1, M2 and M3, when present in the growth medium at concentrations equivalent to gefitinib, were able to exert similar biological effects than gefitinib. As shown in Figure 3C, gefitinib and its metabolites inhibited, in a dose-dependent manner, cell proliferation in sensitive H322 cells with IC_50 _values of 0.13, 0.7, 0.5 and 1.4 μM for gefitinib, M1, M2 and M3 respectively. Figure 3D shows that gefitinib and metabolites inhibited with the same potency EGFR autophosphorylation. These results were further confirmed in both Calu-3 and H292 cell lines.

It should be noted that metabolites were only effective in all the resistant cells at very high concentrations (IC_50 _> 20 μM) (see Figure 3E for H1299) indicating that the metabolites themselves did not have an additive toxic effect (IC_50 _for gefitinib 9 μM).

### Effect of gefitinib on CYP mRNAs expression and EROD activity in NSCLC cell lines

**Figure 4:**
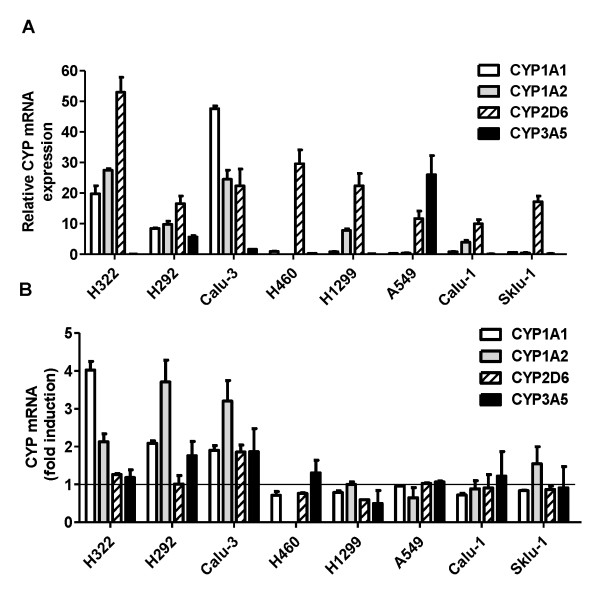
**Effect of gefitinib on CYP mRNAs expression**. A) Basal expression of CYP1A1, 1A2, 2D6 and 3A5 mRNAs in NSCLC cell lines was detected by RT-PCR. The mean values of three independent measurements (± SD) are shown. It is to note that CYP3A4 was undetected. B) The cells were exposed to 0.1 μM gefitinib for 6 h and then gefitinib induction of CYP1A1, 1A2, 2D6 and 3A5 mRNAs was evaluated by RT-PCR and expressed as fold induction relative to basal expression. The mean values of three independent measurements (± SD) are shown.

The baseline transcript levels of CYP1A1, CYP1A2, CYP2D6, CYP3A4 and CYP3A5 were determined in both sensitive and resistant cell lines by RT-PCR and data are summarized in Figure 4A. CYP1A1 and CYP1A2 were expressed at significant levels only in H322, H292 and Calu-3 cell lines, CYP2D6 was detected in all cell lines, whereas CYP3A4 was undetected. CYP3A5 was present at high level only in A549 cells.

The inducibility of individual CYP genes by gefitinib was then investigated and the levels of CYP1A1, CYP1A2, CYP2D6 and CYP3A5 mRNAs were assessed after treating cells with the drug. After 6 h, significantly higher gene expression levels of CYP1A1 and CYP1A2 were observed in all sensitive cell lines. By contrast no significant modulation of gene expression was observed in resistant cell lines (Figure 4B).

In order to evaluate whether modulation of the CYP1A1 transcript levels was associated with changes in the respective enzyme activity levels, we measured the activity of 7-ethoxyresorufin-O-deethylase (EROD), a commonly-used indicator of CYP1A activity [26], both basally and after exposure of cells to gefitinib.

**Figure 5:**
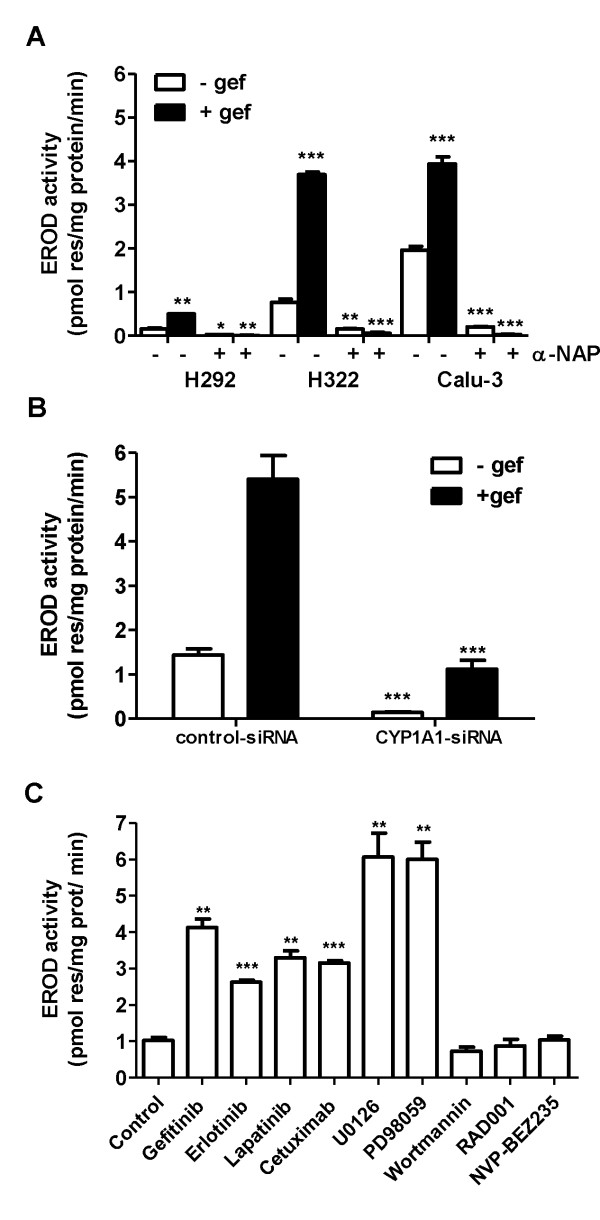
**Effect of gefitinib on EROD activity**. A) EROD assay was performed in intact living cells untreated or treated for 24 h with 0.1 μM gefitinib in the absence or in the presence of 10 μM α-NAP. CYP1A1 activity was measured using 7-ethoxyresorufin as a substrate and expressed as pmol resorufin/mg protein/min. Values given are the means (± SD) of three independent determinations. It is of note that in the gefitinib-resistant cells both the basal and the induced EROD activity were undetectable (not shown) (*P < 0.05; **P < 0.01; ***P < 0.001). B) H322 cells were transfected with siRNA against CYP1A1 or a scrambled negative control with a final concentration of 30 nM for 48 h and then treated for 16 h with 0.1 μM gefitinib before EROD assay was performed. Values given are the means (± SD) of four independent determinations (***P < 0.001). C) EROD assay was performed in intact living cells untreated or treated for 24 h with 0.1 μM gefitinib, 0.1 μM erlotinib, 0.1 μM lapatinib 100 μg/ml cetuximab, 10 μM U0126, 10 μM PD98059, 1 μM Wortmannin, 100 nM RAD001 and 100 nM NVPBEZ235. CYP1A1 activity was measured using 7-ethoxyresorufin as a substrate and expressed as pmol resorufin/mg protein/min. Values given are the means (± SD) of three independent determinations. (**P < 0.01; ***P < 0.001).

In untreated cells, EROD activity was detectable only in sensitive cells, and gefitinib caused a significant increase in this activity (3-6 fold induction) with a maximum at 16-24 h (Figure 5A). Although both CYP1A1 and CYP1A2 carry out EROD activity, the 1A1 form has a much higher specific EROD activity than 1A2 [27]. A further demonstration of CYP1A1 involvement came from the use of 10 μM α-NAP, a CYP1A1 inhibitor [28,29] or from CYP1A1 silencing using siRNAs that significantly inhibited both baseline and gefitinib-induced EROD activity (Figure 5B).

We then tested the effect of other EGFR inhibitors (erlotinib, cetuximab, lapatinib) and of inhibitors of MAPK and PI3K/AKT/mTOR signalling transduction pathways on EROD activity in H322 cell line. As shown in Figure 5C erlotinib, cetuximab and lapatinib induced a significant increase in EROD activity comparable to that induced by gefitinib.

Both MEK inhibitors (PD98059 and U0126) strongly activated CYP1A1 activity, in contrast no increase in the activity was detectable after incubation with the inhibitors of PI3K/AKT/mTOR pathway tested (wortmannin, PI3K inhibitor; RAD001 mTOR inhibitor; NVP-BEZ235 PI3K and mTOR inhibitor)

### Effect of hypoxia, cigarette smoke extract and cell density on gefitinib metabolism

**Figure 6:**
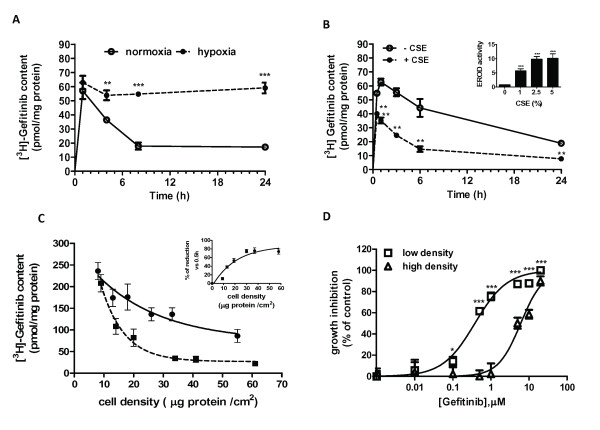
**Conditions affecting gefitinib metabolism: hypoxia, cigarette smoke extract and cell density**. A) H322 cells were incubated with 0.1 μM [^3^H]gefitinib under normoxia or hypoxia for the indicated times. Values given of gefitinib content, expressed as pmol/mg of protein, are the means (± SD) of four independent determinations (**P < 0.01; ***P < 0.001). B) Calu-3 cells were treated for 24 h with 2.5% of CSE and then exposed to 0.1 μM [^3^H]gefitinib for the indicated times. Values given are the means (± SD) of three independent determinations (**P < 0.01; ***P < 0.001). Inset: EROD assay performed in cells untreated or treated for 24 h with 1, 2.5 or 5% CSE expressed as pmol Res/mg protein/min. C) Calu-3 cells were seeded at different cell density (from 7000 to 70000 cells/cm^2^) and after 24 h incubated for 0.5 h (●) or 24 h (■) with 0.1 μM [^3^H]gefitinib. Then, the intracellular [^3^H]gefitinib content was determined and plotted versus final cell density expressed as μg protein/cm^2^. Inset: values are expressed as % of reduction versus 0.5 h value. D) Calu-3 seeded at low and high density (7000 and 70000 cells/cm^2 ^respectively)were exposed for 72 h to different concentrations of gefitinib. Cell growth was assessed using crystal violet staining. Data are expressed as percent inhibition of cell proliferation *versus *control cells. The mean values of three independent measurements (± SD) are shown (*P < 0.05; ***P < 0.001).

Since it is known that hypoxia downregulates the expression and activity of many CYPs including CYP1A1 [30], we evaluated whether hypoxia could prevent gefitinib metabolism and its intracellular loss. The simultaneous exposure of H322 cells to gefitinib and hypoxia (Figure 6A) almost completely prevented gefitinib catabolism inside the cells. Differently, CYP1A1 activity was strongly induced in Calu-3 cells exposed to 2.5% cigarette smoke extract (CSE) for 24 h (see inset Figure 6B) and consequently gefitinib consumption (Figure 6B) was significantly expedited.

Moreover, as expected, cell density strongly affected the reduction in the intracellular level of gefitinib at 24 h in the Calu-3 line (Figure 6C) and consequently cells seeded at high and low density but with a similar growth-rate quotient, exhibited a significant difference in the sensitivity to gefitinib. Indeed, as shown in Figure 6D, cells at low density showed a 15-fold higher sensitivity to gefitinib (IC_50 _0.4 μM) as compared to cells at high density (IC_50 _6 μM).

### Effects of CYP1A1 inhibition on the intracellular level of gefitinib, EGFR autophosphorylation and inhibition of cell growth

**Figure 7:**
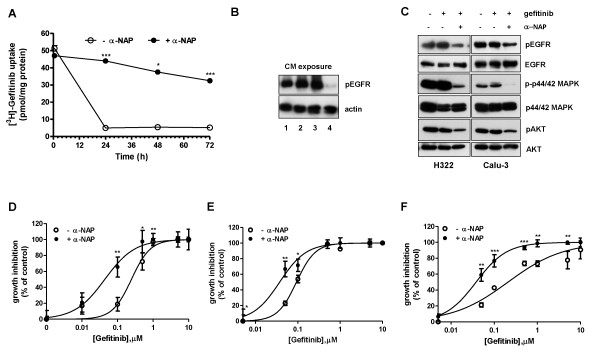
**Effects of CYP1A1 inhibition on intracellular level of gefitinib, EGFR autophosphorylation and inhibition of cell growth**. A) Calu-3 cells were incubated with 0.1 μM [^3^H]gefitinib for 0.5, 24, 48 or 72 h in the absence or in the presence of 10 μM α-NAP. Values given of gefitinib content, expressed as pmol/mg of protein, are the means (± SD) of four independent determinations. α-NAP was renewed after 36 h of gefitinib treatment. (***P < 0.001). B) Calu-3 cells were treated with 0.1 μM gefitinib for 24 h in the absence or in the presence of 10 μM α-NAP, then the conditioned media (CM) were collected and extracts were prepared from H322 cells exposed for 2 h to CM. The effect of CM on EGF (0.1 μg/ml for 5 min)-induced EGFR autophosphorylation was examined by Western blotting using monoclonal antibodies directed against pEGFR(Tyr1068) and actin. The experiment, repeated twice, yielded similar results. Line 1: CM from control cells; line 2: CM from gefitinib treated cells; line 3: CM from α-NAP treated cells; line 4: CM from α-NAP and gefitinib treated cells. C) H322 and Calu-3 cells were incubated for 48 h with 0.1 μM gefitinib in the absence or in the presence of 10 μM α-NAP. Before protein extraction cells were stimulated with 0.1 μg/ml EGF for 5 min. Western blot analysis was performed by using monoclonal antibodies directed against pEGFR(Tyr1068), EGFR, p-p44/42 MAPK, p44/42 MAPK, pAKT (Ser473), AKT. The experiment, repeated three times, yielded similar results. α-NAP was renewed after 24 h of gefitinib treatment. D) Calu-3, (E) H322, (F) H292 were exposed for 72 h to different concentrations of gefitinib in the absence or in the presence of 10 μM α-NAP. Cell growth was assessed using crystal violet staining as described in Materials and Methods. Data are expressed as percent inhibition of cell proliferation *versus *control cells. The mean values of three independent measurements (± SD) are shown. α-NAP was renewed after 36 h of gefitinib treatment (*P < 0.05; **P < 0.01; ***P < 0.001).

In an attempt to better characterize the role of CYP1A1 in sensitive cells, we measured the intracellular content of radiolabeled gefitinib in Calu-3 cells in the presence of 10 μM α-NAP. This inhibitor almost completely abolished the fall in intracellular gefitinib levels after 24 h of treatment (Figure 7A) and the intracellular appearance of the M1 metabolite (not shown).

To further demonstrate that α-NAP was able to maintain a high level of effective drug, Calu-3 cells were treated for 24 h with gefitinib in the presence or absence of α-NAP and then the medium was collected (conditioned media) and extracts from H322 cells exposed to conditioned media for 2 h were prepared to examine the inhibition of EGFR autophosphorylation by Western blot analysis. As shown in Figure 7B in H322 cells EGFR autophosphorylation was unaffected when cells were treated with gefitinib-conditioned medium collected from Calu-3 in the absence of α-NAP, in contrast when the inhibitor was present in the gefitinib-conditioned medium, EGFR autophosphorylation was completely inhibited. These results strongly suggest that in sensitive cells the metabolites released into the medium (low amount of M1 and other undetermined compounds) were ineffective in EGFR inhibition.

The high and constant drug level inside the cells obtained in the presence of α-NAP maintained a significant inhibition of EGFR p44/42 MAPK and AKT phosphorylation even after a prolonged period (48 h) of treatment (Figure 7C) when compared with cells incubated with gefitinib alone.

Sensitive cell lines were then treated with gefitinib in the presence of 10 μM α-NAP for 72 h in order to evaluate the effects of CYP1A1 inhibition on efficacy of gefitinib in inhibiting cell proliferation. In the presence of the inhibitor the IC_50 _for gefitinib, evaluated by crystal violet staining (Figure 7D-F) and confirmed by cell counting and MTT assay (not shown), was reduced 15, 3 and 6 times in Calu-3, H322 and H292 cells respectively.

Overall, these results show that inhibition of CYP1A1 is associated with reduced gefitinib metabolism, increased intracellular gefitinib content and increased drug efficacy in cultured NSCLC cells.

## Discussion

The cytochrome P450 system consists of a large number of enzyme subfamilies involved in the oxidative metabolism of xenobiotics including drugs. They are expressed mainly in the liver, but extra-hepatic expression of a number of these enzymes does occur [31]. Although the primary site of gefitinib metabolism is the liver, tumor cell metabolism can significantly affect treatment effectiveness. However, to our knowledge, no studies have been performed addressing gefitinib metabolism in lung tumor cells.

The present study shows that the drop in gefitinib content observed in EGFR wild-type gefitinib-sensitive cell lines after 24 h of treatment was mainly due to gefitinib metabolism by CYP1A1 activity and not related to a time-dependent modification of influx or efflux processes. Our results indicate that there is a significant difference between gefitinib-sensitive and -resistant cell lines with regard to drug metabolism. Surprisingly, only sensitive cells were able to metabolize gefitinib and as a consequence, after 24 h of treatment, gefitinib disappeared both inside and outside the cells. The majority of radiolabeled gefitinib metabolites were present in the extracellular compartment as not well defined metabolites since we could barely detect the M1 metabolite and M2 or M3 were undetectable. In any case the metabolites present in the medium were not effective in inhibiting EGFR autophosphorylation as demonstrated by the conditioned medium experiment.

It has been reported that both gefitinib and its desmethyl metabolite (M3) formed through CYP2D6, inhibited with a similar potency and selectivity subcellular EGFR tyrosine kinase activity [32]. However, M3 was 15 times less active in a cell-based assay and consequently it was assumed that this metabolite was unlikely to contribute to the activity of gefitinib in vivo due to poor cell penetration.

On the contrary, when metabolites M1, M2 and M3 were tested in our responsive cell models at concentrations equivalent to that of gefitinib, they exhibited a significant inhibition of EGFR autophosphorylation and proliferation in intact cells, indicating their ability to enter cells and to interact with the catalytic domain of EGFR. Finally, in gefitinib resistant cell lines M1, M2 and M3 metabolites were poorly effective (IC_50 _> 20 μM in proliferation assay) indicating that at least these metabolites did not produce additive toxic effects in NSCLC cell lines.

In contrast to its abundant hepatic expression, CYP3A4 seems to play a minor role in lung metabolism, being expressed in only about 20% of cases [33]. Real-time PCR analysis confirmed the lack of expression of this isoform in our NSCLC cell models, as reported for A549 cells [34]. CYP2D6 was detected in all cell lines, whereas both CYP1A1 and CYP1A2 were expressed at significant levels in sensitive cells. Inducibility of CYP1A1 and CYP1A2 transcripts by gefitinib was clearly demonstrated in sensitive cell lines, while induction of CYP1A1 mRNA was not detected in resistant cell lines. EROD activity demonstrated a 3-6 fold induction of CYP1A1 elicited by gefitinib in sensitive cells. To the best of our knowledge, this is the first time that the induction by gefitinib of relevant metabolic enzyme(s) has been demonstrated.

The reason why gefitinib induces CYP expression and activity only in sensitive cells could be ascribed to the ability of gefitinib to inhibit signal transduction pathway downstream EGFR. It has been recently demonstrated that EGF represses the dioxin-mediated induction of CYP1A1 in normal human keratinocytes preventing recruitment of the p300 coactivator [35]. Therefore, EGFR signalling is a repressor of the aryl hydrocarbon receptor and regulates the transcription of numerous genes including CYP1A1. In this context, EGFR inhibitors such as gefitinib, erlotinib, lapatinib or cetuximab might affect the induction of CYP1A1 in those cell types in which the drug effectively inhibits signalling controlled by EGFR. The inhibition of MAPK pathway might represent a link between EGFR inhibition and CYP1A1 induction since PD98059 and U0126, well known MEK1/2 inhibitors, induced CYP1A1 activity as did gefitinib in H322 cells, while none of PI3K/AKT/mTOR inhibitors tested was effective. It is noteworthy that constitutive activation of signaling pathways downstream of EGFR is a recognized mechanism or resistance against reversible EGFR tyrosine kinase inhibitors [5].

We surmise that gefitinib metabolism is a consequence and not the cause of drug responsiveness and might be useful for early evaluation of response to gefitinib in tumor lacking activating mutations.

Since CYP1A1 inducibility strongly correlates with CYP1A1 gene polymorphism [36] we also tested the genotypic asset of our cell lines regarding the two main polymorphic forms of CYP1A1 (CYP1A1*2A and CYP1A1*2C). All the tested cell lines carried a *wild type *homozygous genotype for both the polymorphisms and so we can exclude that different genotypes are involved in the different capability of metabolizing gefitinib.

The role of CYP1A1 polymorphism as a predictor of clinical outcome to EGFR-TKIs in patients with advanced lung cancer has very recently been reported [37]. The authors note that CYP1A1*2A polymorphism correlates with the response to EGFR-TKIs of NSCLC, wild type T/T patients having an improved response of inhibitors versus T/C and C/C alleles.

Studies have shown that the hepatic metabolism of gefitinib is primarily catalyzed via CYP3A4, consequently the effects of known inducers (phenytoin, carbamezepine, rifampicin) and inhibitors (ketoconazole, itraconazole, erythromycin and claritromycin) of CYP3A4 activity have been investigated [6].

Our results indicate that, in NSCLC cells metabolizing gefitinib, CYP1A1 inhibition could lead to increased local exposure to the active drug. In fact, inhibition by α-naphthoflavone was associated with lower gefitinib metabolism and consequently with a prolonged exposure to locally active drug. This leads to enhanced inhibition of EGFR, MAPK and AKT phosphorylation and cell proliferation, with the result of reduced IC_50 _for gefitinib in proliferation assays of EGFR wild-type NSCLC cell lines.

From a medicinal chemistry perspective, these results stress the importance of considering drug pharmacokinetics at the intratumoral cellular level, focusing on the roles of transport and metabolism in the target cells. While the structure of gefitinib makes it a substrate of transporters [9], thus enhancing its activity toward intracellular targets, it also harbors metabolic liabilities in tumor cells. From this point of view, its interaction with CYP3A4 seems mainly related to total-body exposure gefitinib, while CYP1A1 is mainly responsible of its metabolism in tumor cells. A program of structural optimization should thus consider the effects of structure modulation on all these processes in combination.

In addition, a strategy of increasing gefitinib activity by using specific CYP inhibitors, could be pursued in the context of optimizing the use of gefitinib for the treatment of EGFR wild-type gefitinib-sensitive tumors.

Interstitial lung disease (ILD) has been reported as a serious adverse effect of gefitinib treatment [38]. The incidence of acute ILD during gefitinib treatment varies between different ethnic groups occurring more frequently in Japanese patients (4%-6%) than in Caucasian (0.2-0.3%) [39]. Although the precise mechanism of ILD induced by gefitinib remains unknown, it has been proposed that bioactivation of gefitinib by CYP1A1 in the lung may be related to the risk of developing ILD mainly in smokers [8]. In this context the optimisation of CYP1A1 inhibition may not only improve gefitinib efficacy but even reduce the incidence of ILD.

## Abbreviations

CSE: cigarette smoke extract; CYP: cytochrome P450; EGFR: epidermal growth factor receptor; EROD: ethoxyresorufin-O-deethylase; MAPK: mitogen-activated protein kinase; NSCLC: non small cell lung cancer; PI3K/AKT: phosphatidylinositol-3-kinase; TKI: tyrosine kinase inhibitor.

## Competing interests

The authors declare that they have no competing interests.

## Authors' contributions

MG carried out radiolabeled gefitinib experiments, interpreted the results and assisted with the draft of the manuscript; ST carried out EROD experiments; RA carried out LC-MS/MS analysis; PM and GDP carried out RT-PCR experiments; AC and MB carried out Western blot analysis; CF, SLM and EG carried out cell growth experiments; AM, MT, EM and AA critically revised the manuscript; MM performed the statistical analysis; PGP designed the project and assisted with the draft of the manuscript; RRA, analyzed the results and wrote the manuscript. All authors read and approved the final manuscript.
